# Oncological Safety of Testosterone Replacement Therapy in Men With a History of Prostate Cancer: A Systematic Review

**DOI:** 10.7759/cureus.104765

**Published:** 2026-03-06

**Authors:** Vitório A Alexandre Alves, André A Brasileiro Silva, Ernani L Falcao Bisneto, Hiago F Macedo, Gleydson S Lima, Lucas Guilherme P Santos De Souza, Petrônio Eduardo A Barbosa, Porfírio F Medeiros Júnior

**Affiliations:** 1 Department of Urology, Alcides Carneiro University Hospital, Federal University of Campina Grande, Campina Grande, BRA; 2 Department of Urology, Dom Luiz Gonzaga Fernandes Emergency and Trauma Hospital, Campina Grande, BRA

**Keywords:** prostatectomy, prostate-specific antigen, prostatic neoplasms, radiotherapy, testosterone

## Abstract

This systematic review aims to map, critically evaluate, and integrate current evidence on the oncological safety of testosterone replacement therapy (TRT) in men with a history of prostate cancer. For decades, TRT was contraindicated in this population due to the "androgen hypothesis"; however, recent evidence and the "saturation model hypothesis" challenge this dogma. A comprehensive search of seven databases, with no restrictions on publication date, yielded 2,284 studies, of which nine met the inclusion criteria. The reviewed studies, primarily retrospective cohorts and case series, reported that TRT does not appear to increase the risk of biochemical recurrence, disease progression, or mortality in men with low- to intermediate-risk localized prostate cancer following radical prostatectomy, radiotherapy, or under active surveillance. Evidence for high-risk or advanced disease remains limited, and TRT continues to be contraindicated in recurrent or metastatic cases outside of clinical trials. These findings challenge historical restrictions and support the cautious use of TRT in carefully selected patients.

## Introduction and background

The relationship between testosterone and prostate cancer represents one of the most controversial and evolving paradigms in modern medicine. For decades, clinical practice was governed by a dogma that categorically prohibited Testosterone Replacement Therapy (TRT) in men with a history of prostate cancer. This prohibition, however, has been increasingly challenged by a growing body of evidence and by a deeper understanding of androgenic pathophysiology. This systematic review aims to map, critically evaluate, and integrate current evidence on the oncological safety of TRT in this patient population, providing a comprehensive resource to guide clinical decision-making.

The basis for the historical contraindication of TRT in patients with prostate cancer was established in the 1940s by the seminal work of Huggins and Hodges, which earned them the Nobel Prize. They conclusively demonstrated that androgen deprivation, achieved through surgical castration, led to regression of metastatic prostate tumors [1]. This fundamental discovery gave rise to the “androgen hypothesis,” a theory postulating that the development and progression of prostate cancer are driven by androgens in a dose-dependent and linear manner [1]. Under this model, any increase in serum testosterone levels would theoretically be harmful, stimulating the growth of residual or occult cancer cells.

This belief was further reinforced by the observation of the “testosterone flare” phenomenon. Administration of luteinizing hormone-releasing hormone (LHRH) agonists, a cornerstone of androgen deprivation therapy (ADT), causes an initial transient surge in testosterone levels before inducing suppression. This temporary spike was associated with severe adverse events in some patients with advanced disease, solidifying the perception that testosterone elevation was inherently dangerous in the context of prostate cancer. Consequently, TRT was considered an absolute contraindication for any man with a history of the disease.

From the late 20th century onward, the simplistic linear model began to be questioned. Clinical and epidemiological observations failed to demonstrate a consistent association between higher endogenous testosterone levels and increased risk of developing prostate cancer [2]. To reconcile this apparent contradiction, Morgentaler and Traish proposed the “Saturation Model Hypothesis” [3]. This model postulates that androgen receptors (AR) in prostatic tissue become fully saturated at relatively low testosterone levels, typically around 200-250 ng/dL [3].

The main implication of this model is profound: once receptors are saturated, further increases in testosterone levels, such as those occurring when a hypogonadal man is treated with TRT to achieve eugonadal (normal) levels, do not result in significant additional stimulation of prostate tissue growth [3]. According to this model, prostate cancer growth is highly sensitive to variations in testosterone at very low levels (castrate to hypogonadal) but becomes insensitive to changes within the normal physiological range. This concept provided a biologically plausible basis for reconsidering the safety of TRT. However, the model is not without criticism, mainly because it was developed based on animal models of benign prostatic tissue, and its applicability to mutated or advanced prostate cancer cell lines remains an area of active debate [3,4].

Beyond the conceptual framework of the saturation model, recent advances in molecular biology have refined the understanding of androgen receptor (AR) signaling in prostate cancer. Testosterone and dihydrotestosterone (DHT) regulate AR activity through complex mechanisms involving receptor density, ligand affinity, co-regulator recruitment, and chromatin remodeling. Importantly, AR function is dynamically modulated by post-translational modifications, such as phosphorylation, acetylation, and ubiquitination, which influence transcriptional output and tumor behavior [5].

In addition, contemporary research highlights the role of AR amplification, splice variants (e.g., AR-V7), and intratumoral androgen synthesis in sustaining oncogenic signaling even under castrate systemic testosterone levels [6,7]. Novel therapeutic strategies targeting AR degradation pathways, including proteolysis-targeting chimeras (PROTACs), further demonstrate that AR activity depends not solely on circulating testosterone concentration but also on intracellular receptor regulation [8]. These findings reinforce the concept that androgen-prostate cancer interactions are biologically nonlinear and context-dependent.

Moreover, hypogonadism in men with a history of prostate cancer is etiologically heterogeneous. Treatment-induced hypogonadism following androgen deprivation therapy (ADT) produces profound and prolonged suppression of the hypothalamic-pituitary-gonadal axis, with metabolic and skeletal consequences distinct from those observed in age-related or functional hypogonadism [9]. Age-related hypogonadism, frequently associated with obesity, insulin resistance, and chronic inflammation, involves systemic metabolic dysregulation that may independently influence prostate cancer biology [10,11].

The clinical dilemma is pressing. Prostate cancer is one of the most common malignancies in men, and with advances in diagnosis and treatment, the number of survivors is rising significantly. Many of these men suffer from symptomatic hypogonadism, whether due to aging, comorbidities, or as a direct side effect of treatments such as ADT. Hypogonadism negatively impacts quality of life, causing sexual dysfunction, fatigue, loss of muscle mass, decreased bone density, and mood changes [12]. Denying TRT on the basis of historical dogma may unnecessarily deprive these patients of a beneficial therapy. Recent meta-analyses and systematic reviews support a more nuanced perspective, suggesting no clear evidence of increased oncologic risk with TRT in carefully selected men with prostate cancer [2,13]. In addition, new therapeutic concepts such as bipolar androgen therapy illustrate the complexity of androgen-prostate interactions, challenging traditional dogmas and opening avenues for novel treatment strategies [13].

Finally, clinical guidelines have started to acknowledge this paradigm shift. While caution is still advised, recent recommendations from major societies such as the Endocrine Society, American Urological Association (AUA), and the European Association of Urology (EAU) emphasize individualized decision-making regarding TRT in men with a history of prostate cancer [10,14,15]. 

Therefore, the primary aim of this systematic review is to identify, evaluate, and comprehensively integrate the available evidence from observational studies and clinical guidelines regarding the oncological safety of TRT in men with a history of prostate cancer.

## Review

Methodology

Study Design

This is a systematic review conducted in accordance with the 2020 Preferred Reporting Items for Systematic Reviews and Meta-Analyses (PRISMA) statement [16], aiming to synthesize the available evidence on the oncological safety of testosterone replacement therapy in men with a history of prostate cancer. The review protocol was not prospectively registered in PROSPERO or any other database.

Search Strategy

A comprehensive electronic search was performed across seven databases: PubMed, Scopus, EMBASE, ScienceDirect, Cochrane Library, LILACS, and SpringerLink. All records from database inception through September 2025 were eligible for inclusion, with no restrictions on publication date or language. The search strategy combined free-text terms and controlled vocabulary using the MeSH (Medical Subject Headings) system and was applied to titles, abstracts, and keywords. The following Boolean combination was used:

("testosterone replacement therapy" OR "testosterone therapy" OR TRT) AND ("prostate cancer" OR "prostatic neoplasms").

Duplicate records were removed using Rayyan software (Rayyan Systems Inc., Cambridge, MA, US) to ensure each study was counted only once.

Eligibility Criteria

Studies were eligible if they met the predefined PICOS criteria (Population, Intervention, Comparator, Outcomes, and Study Design) [16]. Eligible designs comprised observational studies (cohort studies and case series with more than five patients) and clinical practice guidelines from major urological or endocrinological societies. The population included men with a history of prostate cancer who had undergone definitive therapy or were under active surveillance. The intervention of interest was TRT for symptomatic hypogonadism, and studies were required to report at least one primary oncological outcome (BCR, cancer progression, PCSM, or ACM). Studies were excluded if they lacked oncological outcome data, evaluated TRT for indications other than hypogonadism, or were non-human studies, reviews, case reports with fewer than five patients, or conference abstracts.

Study Selection 

The data extraction and review process was carried out by pairs of independent reviewers using structured instruments and a predetermined consensus process [17,18]. All authors participated in a calibration exercise, in which they independently reviewed a sample paper and then discussed each evaluation item to align understanding and interpretation.

When discrepancies arose during independent assessments, the following consensus strategy was applied [18]: First, reviewers determined whether disagreements stemmed from factual oversights or subjective interpretation. Factual discrepancies, most commonly due to omissions or misreadings, were resolved by rechecking the original manuscript. If reviewers disagreed on the extent to which an item was met, they discussed whether the item had been adequately documented. If disagreement persisted on one point, the lower score was assigned by default. If disagreement exceeded one point, an adjudicator would be involved; however, this was not necessary during the study.

Inclusion Criteria

Studies were considered eligible if they met the following inclusion criteria. Eligible articles included observational studies, such as cohort studies and case series with more than five patients, as well as clinical practice guidelines published by major urological or endocrinological societies. The target population comprised men with a history of prostate cancer, at any stage or grade, who had undergone definitive therapy (radical prostatectomy or radiotherapy) or were under active surveillance. The intervention of interest was the administration of TRT for the management of symptomatic hypogonadism. Studies were required to report at least one of the primary outcomes of interest: biochemical recurrence (BCR), cancer progression, prostate cancer-specific mortality (PCSM), or all-cause mortality (ACM). Only peer-reviewed studies, available from database inception up to September 2025, were considered.

Exclusion Criteria

Studies were excluded if they did not report oncological outcome data. In addition, studies evaluating TRT for indications other than hypogonadism in patients with prostate cancer (e.g., bipolar androgen therapy for castration-resistant disease) were excluded. Further exclusion criteria comprised non-human studies, narrative reviews, systematic reviews, meta-analyses, case reports with fewer than five patients, letters to the editor, technical notes, editorials, conference abstracts, gray literature, and articles without accessible full text.

Methodological Quality and Risk of Bias Assessment 

The methodological quality and risk of bias of the included observational studies were independently assessed by two reviewers using the Newcastle-Ottawa Scale (NOS) [18], a validated tool for this purpose. The NOS employs a point-based scoring system to evaluate study quality across three main domains: selection, comparability between groups, and outcome assessment. Each item can receive one point, with a maximum total of nine points for the highest-quality studies. The specific criteria used to assess cohort studies and case series are detailed in Table 1.

**Table 1:** Evaluation criteria of the Newcastle-Ottawa scale.

Domain	Assessment Item
Selection (Max 4 points)	1. Representativeness of the exposed
	2. Selection of the non-exposed
	3. Ascertainment of exposure (e.g., secure records, structured interviews)
	4. Demonstration that outcome of interest was not present at start of study
Comparability (Max 2 points)	1. Comparability on the basis of design or analysis, controlling for important confounding factors (e.g., Gleason score, tumor stage)
Outcome (Max 3 points)	1. Assessment of outcome (e.g., independent blind assessment, record linkage)
	2. Was follow-up long enough for outcomes to occur
	3. Adequacy follow-up (e.g., lost to follow-up unlikely to introduce bias)

Evaluation Criteria

Based on these criteria, the studies were classified as high, moderate, or low quality. Interobserver agreement was measured using the Kappa (κ) statistic [19]. , and the detailed results for each included study are presented in Table 2.

**Table 2:** Main characteristics of the included studies. RP: Radical Prostatectomy; RT: Radiotherapy; AS: Active Surveillance; BCR: Biochemical Recurrence; PCSM: Prostate Cancer-Specific Mortality; ACM: All-Cause Mortality; N/A: Not Available.

Author (Year)	Country	Study Design	Patient Population	Cancer Risk Profile	No. of Patients (TRT vs. Control)	Median Follow-up (years)	Outcomes Measured
Kaplan-Marans et al. (2024) [20]	United States of America	Retrospective Cohort	Active Surveillance	Low/Intermediate	164 vs. 6,551	4.7 (no TRT) / 5.2 (TRT)	Conversion to Treatment, PCSM, ACM
Ahlering et al. (2020) [21]	United States of America	Retrospective Cohort	Post-RP	Predominantly Low/Intermediate	152 vs. 419	3.5	BCR
Sarkar et al. (2020) [22]	United States of America	Retrospective Cohort	Post-RP & Post-RT	Varied	1,012 vs. 68,972	7.0	BCR, PCSM, ACM
Loeb et al. (2017) [23]	Sweden	Nested Case-Control	General Population	Varied	2,804 (cases) vs. 1,378 (controls)	N/A	Risk of Aggressive Cancer
Kacker et al. (2016) [24]	United States of America	Retrospective Cohort	Active Surveillance	Low/Intermediate	28 vs. 96	3.25	Biopsy Progression
Ory et al. (2016) [25]	United States of America	Case Series	Post-RP, Post-RT, AS	Varied	82 (no control)	3.4	BCR, Progression
Kaplan et al. (2016) [26]	United States of America	Retrospective Cohort	Post-RP/RT	Varied	1,181 vs. 148,173	N/A	ACM, PCSM
Pastuszak et al. (2013) [27]	United States of America	Retrospective Cohort	Post-RP	Low/Intermediate & High	103 vs. 49	2.3	BCR
Sarosdy (2007) [28]	United States of America	Case Series	Post-Brachytherapy	Low/Intermediate	31 (no control)	5.0	BCR

Statistical Analysis

Interobserver agreement was assessed using the Kappa (κ) statistic, computed in BioEstat v5.3 software (BioEstat, Belém, Brazil), following the Landis and Koch scale [19]. The resulting κ-value was 0.865, indicating almost perfect agreement between reviewers.

Results

Study Selection

**Figure 1:**
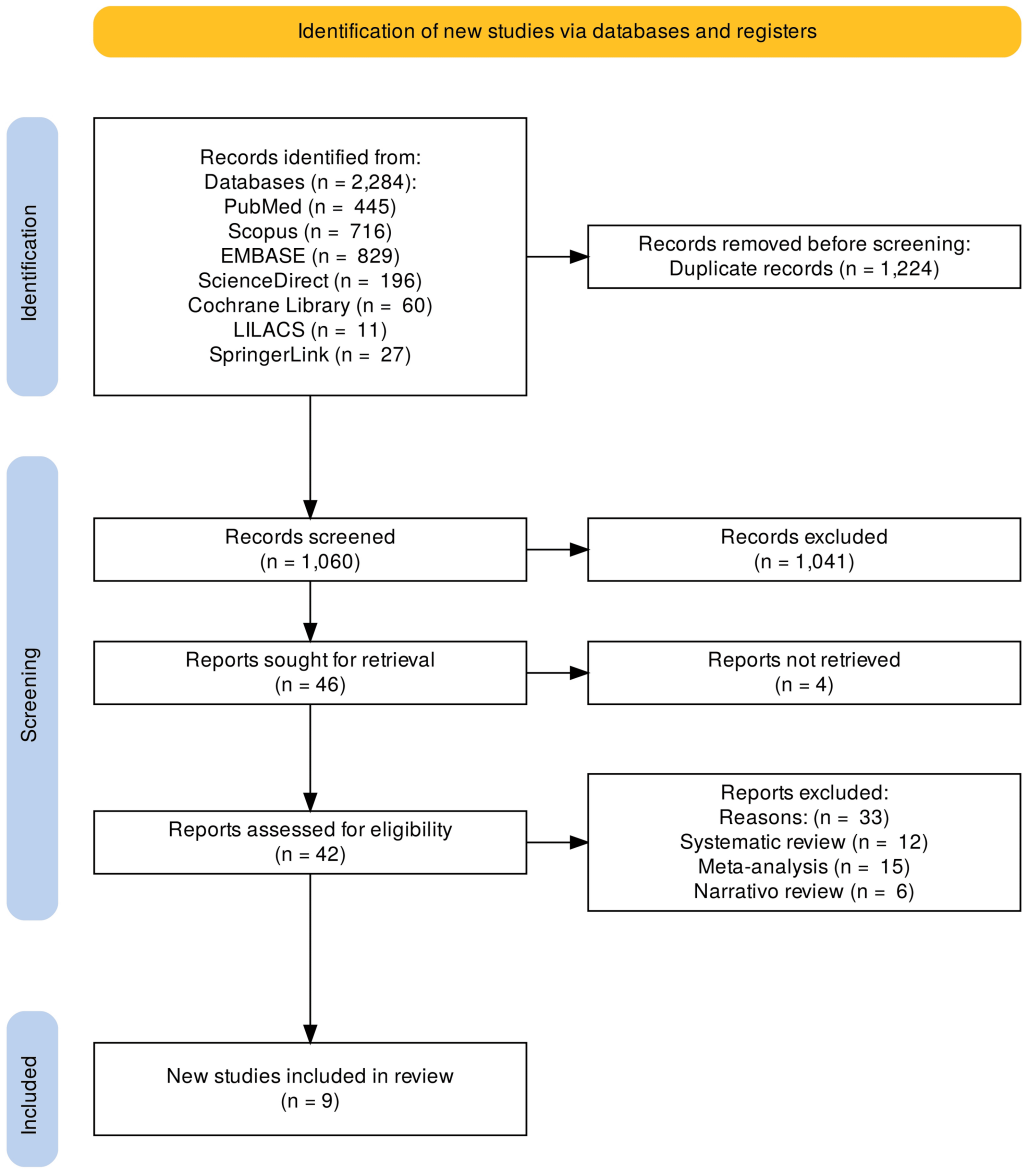
Process of searching and selecting studies for the systematic review, following PRISMA guidelines.

The systematic search initially identified 2,284 articles. After removal of duplicates (n = 1,224) and application of the inclusion and exclusion criteria, 42 full-text articles were assessed for eligibility. Of these, 33 were excluded due to insufficient reporting of oncological outcomes or evaluation of testosterone replacement therapy for indications other than hypogonadism in patients with prostate cancer. A total of nine studies were ultimately included for qualitative synthesis. The study selection process is illustrated in the PRISMA flow diagram (Figure 1).

Study Characteristics 

The main characteristics of the included studies are summarized in Table 2.

Risk of Bias and Study Quality 

Study quality was assessed using the Newcastle-Ottawa Scale (criteria described in Table 3) [18], a validated tool for non-randomized studies. Studies with scores of 7-9 points were considered high quality, those with scores of 5-6 points were classified as moderate quality, and studies scoring below 5 points would be considered low quality. No study scored below five points. Out of the nine studies, seven met the high-quality criteria, and the remaining two were considered of moderate quality due to incomplete methodology reporting or small sample size, indicating a generally low risk of bias among the included observational studies.

**Table 3:** Risk of bias and methodological quality assessment for included studies.

Author (Year)	Selection (*/4)	Comparability (*/2)	Outcome (*/3)	Total Score (*/9)	Quality Rating
Kaplan-Marans et al. (2024) [20]	3	2	3	8	High
Ahlering et al. (2020) [21]	3	2	2	7	High
Sarkar et al. (2020) [22]	3	2	3	8	High
Loeb et al. (2017) [23]	4	2	3	9	High
Kacker et al. (2016) [24]	3	2	2	7	High
Ory et al. (2016) [25]	2	0	3	5	Moderate
Kaplan et al. (2016) [26]	4	2	3	9	High
Pastuszak et al. (2013) [27]	4	2	2	8	High
Sarosdy (2007) [28]	2	0	3	5	Moderate

Results of Individual Studies 

In men managed with radical prostatectomy, Ahlering et al. [21] observed a significantly lower biochemical recurrence (BCR) rate in the TRT group (7.2%) compared to controls (12.6%), suggesting a possible protective effect. Conversely, Sarkar et al. [22], analyzing over 28,000 patients in the Veterans Affairs database, found no difference in BCR or mortality risk. Pastuszak et al. [27] similarly reported comparable recurrence rates between TRT users (3.9%) and controls (16.3%). Among those treated with radiotherapy, Sarkar et al. [22] showed no significant increase in recurrence or mortality with TRT, while Ory et al. [25] reported a 6% BCR rate and Sarosdy [28] documented no recurrences after brachytherapy over a 5-year follow-up. In the active surveillance cohort, Kaplan-Marans et al. [20] found a lower rate of conversion to definitive treatment among TRT users (HR 0.66), although this likely reflected selection bias. Kaplan et al. [26] found no association between TRT use and prostate cancer-specific mortality (PCSM) or all-cause mortality (ACM). Kacker et al. [24] reported no significant difference in biopsy progression between the TRT and control groups. From the population-based registry in Sweden, Loeb et al. [23] noted an inverse association between TRT and aggressive prostate cancer diagnosis (OR 0.50), a finding attributed to detection bias.

Comparative Analysis of Clinical Practice Guidelines

The interpretation of available evidence by different professional bodies has resulted in notably discordant clinical practice guidelines, as shown in Table 4, creating uncertainty for clinicians and patients.

**Table 4:** Comparison of clinical practice guideline recommendations on TRT after prostate cancer. AUA: American Urological Association; EAU: European Association of Urology; RP: Radical Prostatectomy; RT: Radiotherapy; AS: Active Surveillance; PSA: Prostate-Specific Antigen; N/A: Not Applicable; RCT: Randomized Clinical Trial.

Guideline (Year)	Recommendation for Post-RP/RT Patients	Recommendation for AS Patients	Specific Criteria for Use	Rationale/Level of Evidence
Endocrine Society (2018) [10]	Recommends against	Recommends against	N/A (contraindicated)	Theoretical risk of cancer stimulation; absence of long-term RCT data
AUA (2018) [14]	May be considered	Inadequate evidence; shared decision	Favorable pathology (post-RP), undetectable PSA	Expert Opinion / Limited Evidence
EAU (2025) [15]	May be offered with caution	Not addressed directly (treatment requires absence of active disease)	Low risk of recurrent prostate cancer; at least 1 year of follow-up; PSA < 0.01 ng/ml	Weak recommendation / Poor evidence quality (lack of sufficient long-term safety data)

Synthesis of Results

Across the nine included studies, TRT was not linked to increased biochemical recurrence, disease progression, or mortality in men with a history of localized prostate cancer [20-28], as summarized in Table 5. BCR rates after surgery or radiotherapy ranged from 0% to 12.6% in TRT users, comparable to or lower than those of controls. In active surveillance, progression and conversion rates were not significantly different between groups. The most consistent finding is the oncological safety of TRT in low- to intermediate-risk patients, with neutral or favorable outcomes across retrospective cohorts and large database studies. Evidence in high-risk or advanced disease is sparse and of low quality, with some reports of PSA increases and progression, supporting current guideline recommendations against TRT in this population [10,14,15].

**Table 5:** Summary of oncologic outcomes by patient subgroup and treatment history. HR: Hazard Ratio; PCSM: Prostate Cancer-Specific Mortality; ACM: All-Cause Mortality; TRT: Testosterone Replacement Therapy.

Patient Subgroup	Author (Year)	Biochemical Recurrence (BCR) Outcome	Mortality (PCSM & ACM) Outcome	Overall Certainty of Evidence
Post-Radical Prostatectomy	Ahlering et al. (2020) [21], Sarkar et al. (2020) [22], Pastuszak et al. (2013) [27]	No increased risk (HR ~1.07); possible risk reduction (HR ~0.54) in selected cohorts.	No increased risk of PCSM or ACM.	Moderate
Post-Radiotherapy	Sarkar et al. (2020) [22], Ory et al. (2016) [25], Sarosdy et al. (2007) [28]	No increased risk (HR ~1.07); low BCR rates (0-6%).	No increased risk of PCSM or ACM.	Moderate
Active Surveillance	Kaplan-Marans et al. (2024) [20], Kacker et al. (2016) [24]	No increased risk of conversion to treatment or biopsy progression.	No prostate cancer deaths in TRT group; no increase in ACM.	Low to Moderate
High-Risk/Advanced Disease	Loeb et al. (2017) [23], Kaplan et al. (2016) [26]	Very low-quality evidence; concern for PSA increase and progression.	Unknown risk; contraindicated by guidelines.	Very Low

Discussion 

Biochemical Recurrence (BCR)

Radical prostatectomy (RP) provides an ideal scenario for assessing recurrence, as prostate-specific antigen (PSA) is expected to become undetectable after complete removal of prostatic tissue. Therefore, any subsequent rise serves as a highly sensitive marker of BCR. However, persistently detectable PSA levels after surgery may occur in the presence of residual disease, including occult metastatic foci not identified on conventional preoperative imaging.

The literature on TRT after RP is extensive but yields heterogeneous results. The most robust evidence comes from a Veterans Affairs (VA) health system analysis of 28,651 men, which demonstrated no significant difference in BCR risk between TRT users and non-users (HR 1.07; 95% CI 0.84-1.36) [22]. This large, population-based study strongly suggests that TRT does not increase recurrence risk.

Smaller single-center studies report seemingly more favorable outcomes. For example, Ahlering et al. [21] found a lower recurrence rate among TRT users (7.2%) compared with controls (12.6%), and TRT remained an independent predictor of recurrence-free survival. Pastuszak et al. [27] also observed numerically fewer recurrences in TRT users, though differences were not statistically significant. While these findings appear to suggest a protective effect, the most plausible explanation is selection bias: men chosen for TRT are typically healthier, have more favorable tumor characteristics, and receive closer follow-up, all of which contribute to improved outcomes. Biologically, a “protective” effect is implausible under the saturation model, which predicts neutrality rather than benefit. Therefore, the most defensible interpretation is non-inferiority (safety), not superiority.

After radiotherapy (RT), recurrence assessment is more complex, as the prostate remains in situ and continues to secrete PSA. Nevertheless, the VA cohort, including 41,333 men, again found no increase in BCR with TRT (HR 1.07; 95% CI 0.90-1.27) [22]. Smaller case series report similarly low recurrence rates, including Sarosdy [28], who observed no events after brachytherapy, and Ory et al. [25], who reported a 6% recurrence rate after RT. Together, these data reinforce the conclusion that TRT does not elevate recurrence risk following local therapy.

Cancer Progression

In men managed with active surveillance (AS), the concern is that TRT could accelerate the progression of indolent tumors. The largest study to date, Kaplan-Marans et al. [20], analyzed 6,715 men and found a lower rate of conversion to active treatment in TRT users. While provocative, this result is best understood as an artifact of selection bias: men deemed eligible for TRT likely had more favorable disease at baseline and more consistent follow-up. Smaller studies, such as Kacker et al. [24], reported no significant difference in biopsy progression between TRT and controls. Collectively, these data suggest that TRT does not accelerate disease progression in carefully selected men on AS, but they do not justify the interpretation of a protective effect.

In high-risk or advanced disease, the evidence remains scarce and low quality. A meta-analysis suggested low recurrence rates [4], but the certainty of evidence is poor. Some studies report PSA rises with TRT in this population, emphasizing the need for extreme caution [26]. Importantly, a Swedish registry study suggested a lower risk of aggressive cancers among TRT users [23], but this likely reflects detection bias, as these men are more closely monitored.

TRT in men with recurrent or metastatic disease remains contraindicated outside clinical trials [26,27]. In this setting, the saturation model may not apply, and historical concepts of androgen-driven growth regain relevance. Small case series reporting PSA and radiographic progression support maintaining TRT as unsafe in advanced disease.

It is also essential to distinguish TRT from Bipolar Androgen Therapy (BAT), which uses supraphysiological testosterone as an experimental treatment in castration-resistant prostate cancer (CRPC). BAT is a therapeutic strategy, not a replacement approach for hypogonadal symptoms [14].

Mortality

Data on survival endpoints are consistent and reassuring. The VA analysis found no increase in prostate cancer-specific mortality (PCSM) or all-cause mortality (ACM) after TRT in men treated with RP or RT [22]. Kaplan et al. [26], analyzing nearly 150,000 survivors in SEER-Medicare, similarly found no association between TRT and either PCSM or ACM. In AS, Kaplan-Marans et al. [20] reported no prostate cancer-specific deaths among TRT users. These converging results across large datasets strengthen the conclusion that TRT does not compromise survival in appropriately selected patients.

Limitations

This review is limited by methodological heterogeneity among the included studies, particularly regarding patient populations, definitions of biochemical recurrence (BCR), TRT regimens, and follow-up durations. Additionally, the absence of prospective, long-term randomized clinical trials and the absence of meta-analysis limit the generalizability and quantitative strength of the conclusions.

## Conclusions

Testosterone replacement therapy (TRT) appears to be oncologically safe in carefully selected men with low- to intermediate-risk prostate cancer, including those treated with radical prostatectomy, radiotherapy, or managed with active surveillance. Based on this review, we recommend that TRT be considered only in symptomatic hypogonadal patients who meet stringent selection criteria and are closely monitored with regular PSA assessments. Clinical decision-making should incorporate patient risk stratification, tumor characteristics, and comorbidities to minimize potential adverse outcomes. Awareness of the limitations of existing evidence, particularly selection and detection biases, is essential to avoid misinterpretation of apparent protective effects. Incorporating these considerations into clinical practice can allow safe management of hypogonadism with TRT while maintaining oncological vigilance.
